# A rare case of extremely high counts of circulating tumor cells detected in a patient with an oral squamous cell carcinoma

**DOI:** 10.1186/s12885-016-2591-8

**Published:** 2016-07-27

**Authors:** Xianglei Wu, Romina Mastronicola, Qian Tu, Gilbert Charles Faure, Marcelo De Carvalho Bittencourt, Gilles Dolivet

**Affiliations:** 1 Laboratory of Immunology, Nancytomique platform, CHRU of Nancy, rue du Morvan, 54500 Vandoeuvre-lès-Nancy, France; 2 SBS Department, CRAN, UMR 7039 CNRS, University of Lorraine, Avenue de la Forêt de Haye, 54500 Vandoeuvre-lès-Nancy, France; 3 Department of Otorhinolaryngology - Head and Neck surgery, Zhongnan Hospital of Wuhan University, No. 169 Donghu Road, 430071 Wuhan, China; 4 Head and Neck Surgery and Dental Units, Oncologic Surgery Department, Institut de Cancérologie de Lorraine, 6 Avenue de Bourgogne, 54500 Vandœuvre-lès-Nancy, France

**Keywords:** Oral squamous cell carcinoma, Circulating tumor cells, Head and neck squamous cell carcinoma, Survival

## Abstract

**Background:**

Despite aggressive regimens, the clinical outcome of head and neck squamous cell carcinoma remains poor. The detection of circulating tumor cells could potentially improve the management of patients with disseminated cancer, including diagnosis, treatment strategies, and surveillance. Currently, CellSearch^®^ is the most widely used and the only Food and Drug Administration-cleared system for circulating tumor cells detection in patients with metastatic breast, colorectal, or prostate cancer. In most cases of head and neck squamous cell carcinoma, only low counts of circulating tumor cells have been reported.

**Case presentation:**

A 56-year-old white male with no particular medical history, was diagnosed with a squamous cell carcinoma of oral cavity. According to the imaging results (computed tomography and ^18^F-fluorodeoxyglucose positron emission tomography / computed tomography) and panendoscopy, the TNM staging was classified as T4N2M0. A non-interruptive pelvimandibulectomy was conducted according to the multidisciplinary meeting advices and the postoperative observations were normal. The patient complained of a painful cervical edema and a trismus 6 weeks after the surgery. A relapse was found by computed tomography and the patient died two weeks later. The search for circulating tumor cells in peripheral venous blood by using the CellSearch^®^ system revealed a very high count compared with published reports at three time points (pre-operative: 400; intra-operative: 150 and post-operative day 7: 1400 circulating tumor cells). Of note, all detected circulating tumor cells were epidermal growth factor receptor negative.

**Conclusion:**

We report here for the first time a rare case of oral squamous cell carcinoma with extremely high circulating tumor cells counts using the CellSearch^®^ system. The absolute number of circulating tumor cells might predict a particular phase of cancer development as well as a poor survival, potentially contributing to a personalized healthcare.

## Background

Head and neck cancer, the sixth leading cancer by incidence worldwide [1], develops from the mucosal linings of the upper aerodigestive tract. Approximately 95 % of head and neck cancers have a squamous cell histological aspect [2], also known as HNSCC (head and neck squamous cell carcinoma). Despite aggressive treatment regimens, many HNSCC patients (20–30 %) develop locoregional recurrence (local recurrence / cervical lymph node metastasis) or distant metastases. In addition, even in cases where the resection margins were negative in histopathological examination, there is still a risk of local recurrence in 20 % of cases [3, 4]. Consequently, the five-year survival rate for all stages combined is 40–50 %. Of note, human papillomavirus (HPV)-positive HNSCC patients have a significantly better prognosis than HPV-negative patients, for instance lower disease specific mortality and recurrence rate [5].

Circulating tumor cells (CTC), which eventually detach from the primary tumor and disseminate in the blood and other body fluids, have been considered as a “liquid biopsy” in the clinical assessment of cancer patients [6, 7]. The diagnostic and prognostic values of CTC detection have been established in some cancers, like breast, colorectal and prostate cancers [8–10], but not yet in HNSCC. Recent studies in HNSCC with small patient cohorts have shown the potential clinical utility of CTC [11]. However, further evidence is needed for evaluating comprehensively its application, such as unusual cases. Here, we report extremely high enumerations of CTC detected in a patient with an oral squamous carcinoma, which might also explain his quick relapse and short survival.

## Case presentation

A 56-year-old white male, former pipefitter, 68 kg /169 cm, with no particular personal or familiar medical-surgical history, presented with a lesion of oral cavity of recent apparition. The cancer risk factors included unweaned smoking, valued at about 40 pack-years, and alcohol consumption (weaned for 3 years). Physical examination revealed an ulcerative lesion on the right anterolateral floor of oral cavity, which was adherent to the gingival fibro mucosa. Several leukoplakias were also observed on the gingival mucosa. The rest of the otorhinolaryngology examination was unremarkable.

**Fig. 1:**
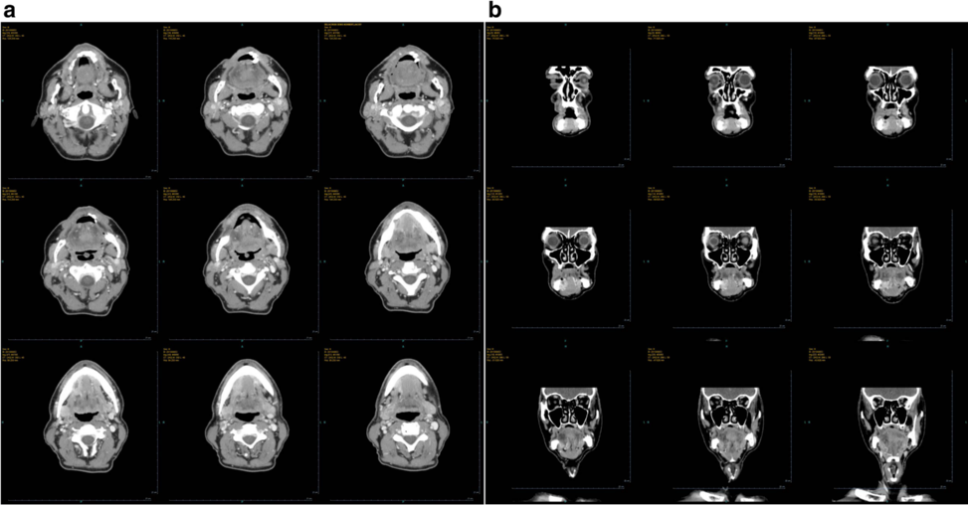
Preoperative CT images. 1a axial plane; 1b coronal plane

**Fig. 2:**
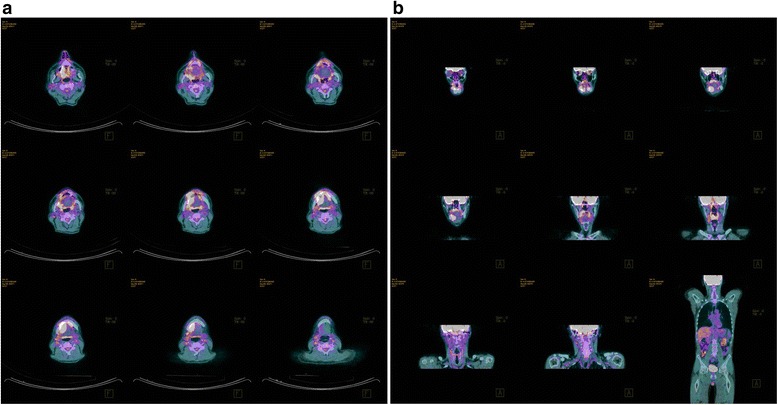
Preoperative ^18^F-FDG PET/CT. 2a axial plane; 2b coronal plane

A biopsy confirmed the lesion as an invasive well- / moderately-differentiated squamous cell carcinoma. A contrasted computer tomography (CT) scan showed a tumoral process involving the muscles of the anterolateral floor of oral cavity, which extended about 3 cm in the long axis and remained lateralized to the right (Fig. 1). The lesion was in contact with the mandible and furthermore developed a suspicious bone notch. No cervical lymph nodes of significant size and no other suspicious lesions on the cervical-thoracic level were present in this CT scan. A ^18^F-fluorodeoxyglucose positron emission tomography / computed tomography (^18^F-FDG PET/CT) scan found a lesion of intense hypermetabolism next to the right genio-glosse triangular muscles and lips, which seemed to repel the omohyoid muscle without infiltrating it (Fig. 2). A lytic aspect of the cortical bones of the mandible body suggested a bone extension. ^18^F-FDG uptakes were perceptible in lymph nodes of right groups Ib and II. The patient received a panendoscopy under general anesthesia. The upper gastrointestinal endoscopy and the bronchoscopy did not find any abnormalities.

**Fig. 3:**
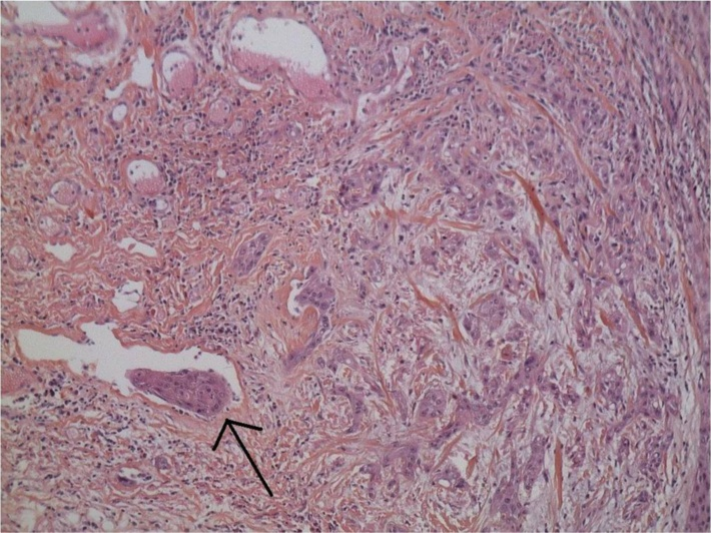
Fixed HE-stained pathology of excised tissue (original magnification 20×). The tissue corresponds to an infiltrative poorly-differentiated squamous cell carcinoma. Arrow indicates an embolus in the vessel

The TNM staging was classified as T4N2M0 (according to American Joint Committee on Cancer 2009) [12] and noninterruptive pelvimandibulectomy was validated as the primary treatment by a multidisciplinary meeting. The surgery was performed in a satisfactory manner 2 months after the first consultation. The post-operative care was performed by standard procedures without abnormalities. The pathological analysis of excised specimens confirmed the squamous cell carcinoma histology as well as the lymph node metastases (Fig. 3), suggesting the definitive TNM stage as pT4aN2cM0. In addition, the margins were negative but multiple tumor nodules were found in the muscle. Immunohistochemical analysis for HPV showed the staining of p40 but no expression of p16.

**Fig. 4:**
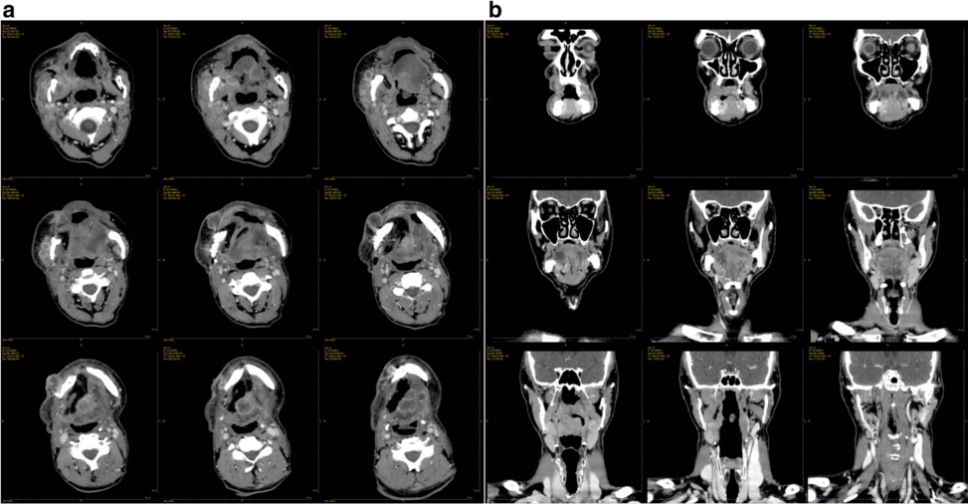
Postoperative CT images. 2a axial plane; 2b coronal plane

At the end of 6 weeks postoperative follow-up, the patient complained of a painful cervical edema as well as a trismus. A CT scan was ordered, which found regional multiple recurrences (Fig. 4). A multidisciplinary meeting updated the treatment strategy including a surgical retake, followed by radiochemotherapy. However, the patient died 2 weeks later due to cancer related complications.

### Detection of CTC

**Fig. 5:**
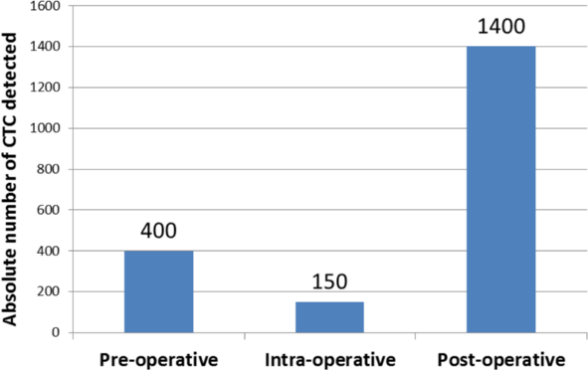
CTC enumeration per time point

**Fig. 6:**
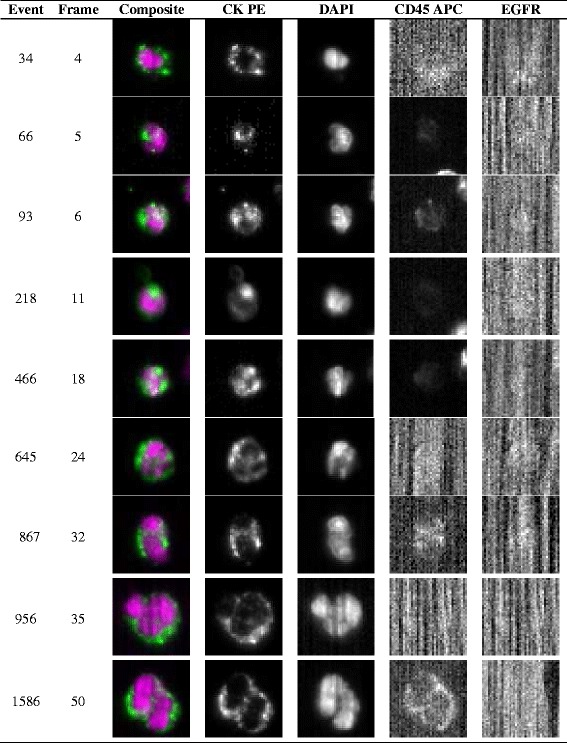
Representative CTC images from the CellSearch^®^ system. Representative pre-operative CTC images of 7.5 ml blood from the patient with an oral squamous cell carcinoma. DAPI, 4′, 6-diamidino-2-phenylindole; CK-PE, cytokeratin-phycoerythrin; APC, allophycocyanin

The patient was recruited into a clinical research program (VADS - EudraCT N°: 2010-A00586-33, approved by the regional ethic committee “Comité de Protection des Personnes Est III”), which aimed to evaluate the prognostic value of CTC in HNSCC. Venous blood samples were collected at three time points (preoperative day-1, intraoperative, and postoperative day 7) for the detection of CTC. The manipulations were performed by using the CellSearch^®^ system (Veridex LLC, Raritan, NJ) according to a standard protocol [13]. The commercially available CellSearch^®^ Tumor Phenotyping Reagent Epidermal Growth Factor Receptor (EGFR) kit (Veridex LLC, Raritan, NJ, USA) was used on the fourth channel of fluorescence of the CellSearch^®^ system following the manufacturer’s instructions. The pre-, intra-, and post-operative enumerations of CTC are shown in Fig. 5. A high count of CTC was already detected at baseline (400 CTC), decreasing by 67.5 % at the intra-operative time point (150 CTC), after which it increased significantly (1400 CTC). In particular, all CTC were EGFR negative. Typical images of CTC are shown in Fig. 6.

## Discussion

To our knowledge, this is the first report of extremely high numbers of CTC in a case of squamous cell carcinoma of oral cavity. At present, the CellSearch^®^ System is the most commonly used and the only Food and Drug Administration (FDA)-cleared CTC detection technique, working on an EpCAM (Epithelial cell adhesion molecule) based-capture enrichment. Current published data on CTC count using the CellSearch^®^ System in HNSCC patients reported a range of 0–5 CTC in 7.5 ml blood, associated with the clinical features or survival [14–18]. In the case reported here, the initial CTC count was already dozens of times higher than the known published maximum. The CTC count achieved even several hundred numbers at post-operative day 7. Moreover, as a label-dependent approach, the Cellsearch® might have underestimated actual CTC load in circulation owing to the possible existence of EpCAM negative tumor cells.

Based on the fact that the absolute number of EpCAM positive circulating epithelial cells was upregulated and were negative for EGFR expression, one can hypothesize that the CTC were probably in a particular phase associated with mesenchymal–epithelial transition (MET). Since the samples were detected individually according to the standard semi-automated procedure, we can exclude the possibility of technical confounders, such as contaminations. Besides, studies using the same method in other malignancies, like breast [19], prostate [20], colorectal [21], and gastrointestinal cancer [22] have rarely found such high CTC counts. EGFR is overexpressed in over 90 % of HNSCC [23] but with a huge discordance between the primary tumor and CTC. Grisanti et al. [17] found that EGFR was expressed in 45 % of CTC from patients with recurrent or metastatic HNSCC. In this work, no CTC was found to be EGFR positive regardless of time point. Generally, MET is thought as the reverse biological process of epithelial-mesenchymal transition (EMT), but relatively poor evidence has been found about its role in cancer when compared to the extensive studies of the latter. Given that MET induce upregulation of epithelial markers (E.g. EpCAM) and that MET facilitates allowing cancerous cells to regain epithelial properties, the absence of EpCAM + EGFR + CTC vis-a-vis the enormous amount of EpCAM + EGFR-CTC might suggest the development of MET. Genetic studies revealed that mutations of the EGFR gene family correlate with mRNA abundance and protein level in patients with HNSCC [24, 25]. Thus, in the case reported here, the lack of EGFR expression on CTC could be also related to a rare mutated tumor type. In addition, the higher count of CTC at the post-operative day 7 could be due to the re-entry of disseminative tumor cells in mesenchymal tissues into the circulation [26].

The very short survival of this patient (disease-free survival, DFS and overall survival, OS) might be associated with the high absolute number of CTC. Even if the patient was diagnosed at stage IVA, the DFS of 1 month and the OS of 2 months still indicate a rapid progression. The routine evaluation, TNM staging, seemingly could not provide a plausible prediction of this clinical outcome. Of note, the presence of CTC has been reported to be an independent prognostic factor for predicting survival of HNSCC patients with higher sensitivity at various disease stages than routine staging procedures [17, 27, 28]. High post-operative levels of CTC have also been reported to accurately predict tumor recurrence [29]

## Conclusion

In conclusion, this is the first report of a rare case of extremely high CTC counts potentially associated with the short survival in the oral squamous cell carcinoma setting. The CTC detection constantly monitors the tumor biology changes as a “liquid biopsy”. We believe that such findings are of great significance in terms of personalized healthcare, although might not be frequent. Our data also might suggest a conceivable observation of MET, which requires confirmation by additional specific studies.

## Abbreviations

^18^F-FDG PET/CT, 18 F-fluorodeoxyglucose positron emission tomography / computed tomography; CT, computed tomography; CTC, circulating tumor cells; DFS, disease-free survival; EGFR, epidermal growth factor receptor; EMT, epithelial-mesenchymal transition; EpCAM, epithelial cell adhesion molecule; FDA, food and drug administration; HNSCC, head and neck squamous cell carcinoma; HPV, human papillomavirus; MET, mesenchymal-epithelial transition; OS, overall survival; TNM, tumor nodes metastasis

## References

[1] Siegel RL, Miller KD, Jemal A (2015). Cancer statistics, 2015. CA Cancer J Clin.

[2] Chaturvedi AK, Anderson WF, Lortet-Tieulent J, Curado MP, Ferlay J, Franceschi S, Rosenberg PS, Bray F, Gillison ML (2013). Worldwide trends in incidence rates for oral cavity and oropharyngeal cancers. J Clin Oncol.

[3] Looser KG, Shah JP, Strong EW (1978). The significance of “positive” margins in surgically resected epidermoid carcinomas. Head Neck Surg.

[4] Spiro RH, Guillamondegui O, Paulino AF, Huvos AG (1999). Pattern of invasion and margin assessment in patients with oral tongue cancer. Head Neck.

[5] O’Rorkea MA, Ellisonb MV, Murraya LJ, Moranc M, Jamesc J, Anderson LA (2012). Human papillomavirus related head and neck cancer survival: a systematic review and meta-analysis. Oral Oncol.

[6] Alix-Panabieres C, Pantel K (2013). Circulating tumor cells: liquid biopsy of cancer. Clin Chem.

[7] Schmidt H, Kulasinghe A, Perry C, Nelson C, Punyadeera C (2016). A liquid biopsy for head and neck cancers. Expert Rev Mol Diagn.

[8] Cohen SJ, Punt CJ, Iannotti N, Saidman BH, Sabbath KD, Gabrail NY, Picus J, Morse M, Mitchell E, Miller MC (2008). Relationship of circulating tumor cells to tumor response, progression-free survival, and overall survival in patients with metastatic colorectal cancer. J Clin Oncol.

[9] Cristofanilli M, Hayes DF, Budd GT, Ellis MJ, Stopeck A, Reuben JM, Doyle GV, Matera J, Allard WJ, Miller MC (2005). Circulating tumor cells: a novel prognostic factor for newly diagnosed metastatic breast cancer. J Clin Oncol.

[10] Danila DC, Heller G, Gignac GA, Gonzalez-Espinoza R, Anand A, Tanaka E, Lilja H, Schwartz L, Larson S, Fleisher M (2007). Circulating tumor cell number and prognosis in progressive castration-resistant prostate cancer. Clin Cancer Res.

[11] Kulasinghe A, Perry C, Jovanovic L, Nelson C, Punyadeera C (2015). Circulating tumour cells in metastatic head and neck cancers. Int J Cancer.

[12] Edge SB, Compton CC (2010). The American Joint Committee on Cancer: the 7th edition of the AJCC cancer staging manual and the future of TNM. Ann Surg Oncol.

[13] Janssen Diagnostics L. CELLSEARCH® Circulating Tumor Cell Epithelial Kit (IVD) IFU No. e631600001-V 2013-08. 2013.

[14] Nichols AC, Lowes LE, Szeto CC, Basmaji J, Dhaliwal S, Chapeskie C, Todorovic B, Read N, Venkatesan V, Hammond A (2012). Detection of circulating tumor cells in advanced head and neck cancer using the Cell Search system. Head Neck.

[15] Bozec A, Ilie M, Dassonville O, Long E, Poissonnet G, Santini J, Chamorey E, Ettaiche M, Chauviere D, Peyrade F (2013). Significance of circulating tumor cell detection using the Cell Search system in patients with locally advanced head and neck squamous cell carcinoma. Eur Arch Otorhinolaryngol.

[16] Grobe A, Blessmann M, Hanken H, Friedrich RE, Schon G, Wikner J, Effenberger KE, Kluwe L, Heiland M, Pantel K (2014). Prognostic relevance of circulating tumor cells in blood and disseminated tumor cells in bone marrow of patients with squamous cell carcinoma of the oral cavity. Clin Cancer Res.

[17] Grisanti S, Almici C, Consoli F, Buglione M, Verardi R, Bolzoni-Villaret A, Bianchetti A, Ciccarese C, Mangoni M, Ferrari L (2014). Circulating tumor cells in patients with recurrent or metastatic head and neck carcinoma: prognostic and predictive significance. PLoS One.

[18] Buglione M, Grisanti S, Almici C, Mangoni M, Polli C, Consoli F, Verardi R, Costa L, Paiar F, Pasinetti N (2012). Circulating tumour cells in locally advanced head and neck cancer: preliminary report about their possible role in predicting response to non-surgical treatment and survival. Eur J Cancer.

[19] Zhang L, Riethdorf S, Wu G, Wang T, Yang K, Peng G, Liu J, Pantel K (2012). Meta-analysis of the prognostic value of circulating tumor cells in breast cancer. Clin Cancer Res.

[20] Moreno JG, O’Hara SM, Gross S, Doyle G, Fritsche H, Gomella LG, Terstappen LW (2001). Changes in circulating carcinoma cells in patients with metastatic prostate cancer correlate with disease status. Urology.

[21] Negin BP, Cohen SJ (2010). Circulating tumor cells in colorectal cancer: past, present, and future challenges. Curr Treat Options Oncol.

[22] Hiraiwa K, Takeuchi H, Hasegawa H, Saikawa Y, Suda K, Ando T, Kumagai K, Irino T, Yoshikawa T, Matsuda S (2008). Clinical significance of circulating tumor cells in blood from patients with gastrointestinal cancers. Ann Surg Oncol.

[23] Rubin Grandis J, Melhem MF, Gooding WE, Day R, Holst VA, Wagener MM, Drenning SD, Tweardy DJ (1998). Levels of TGF-alpha and EGFR protein in head and neck squamous cell carcinoma and patient survival. J Natl Cancer Inst.

[24] Nakazaki K, Kato Y, Taguchi T, Inayama Y, Ishiguro Y, Kondo N, Horiuchi C, Sakakibara A, Tsukuda M (2010). Heterozygous mutation (G/G → G/A) at nt 2607 of the EGFR gene is closely associated with increases in EGFR copy number and mRNA half life, but impaired EGFR protein synthesis in squamous cell carcinomas of the head and neck–implication for gefitinib efficacy. Oncol Lett.

[25] Hama T, Yuza Y, Suda T, Saito Y, Norizoe C, Kato T, Moriyama H, Urashima M (2012). Functional mutation analysis of EGFR family genes and corresponding lymph node metastases in head and neck squamous cell carcinoma. Clin Exp Metastasis.

[26] van Dalum G, van der Stam GJ, Tibbe AG, Franken B, Mastboom WJ, Vermes I, de Groot MR, Terstappen LW (2015). Circulating tumor cells before and during follow-up after breast cancer surgery. Int J Oncol.

[27] Jatana KR, Balasubramanian P, Lang JC, Yang L, Jatana CA, White E, Agrawal A, Ozer E, Schuller DE, Teknos TN (2010). Significance of circulating tumor cells in patients with squamous cell carcinoma of the head and neck: initial results. Arch Otolaryngol Head Neck Surg.

[28] Tinhofer I, Konschak R, Stromberger C, Raguse JD, Dreyer JH, Johrens K, Keilholz U, Budach V (2014). Detection of circulating tumor cells for prediction of recurrence after adjuvant chemoradiation in locally advanced squamous cell carcinoma of the head and neck. Ann Oncol.

[29] Galizia G, Gemei M, Orditura M, Romano C, Zamboli A, Castellano P, Mabilia A, Auricchio A, De Vita F, Del Vecchio L (2013). Postoperative detection of circulating tumor cells predicts tumor recurrence in colorectal cancer patients. J Gastrointest Surg.

